# Rheumatic Immune-Related Adverse Events: Immune Checkpoint Inhibitor Therapy and Ramifications for Health Disparities

**DOI:** 10.7759/cureus.90585

**Published:** 2025-08-20

**Authors:** Laura Kobashigawa, Nicole Zagelbaum, Katherine Ruddy, Martha Delgado, Baljeet Rai, Victoria Gevorgyan, Kiana Mortezaei, William Stohl, Elizabeth Taylor-Albert

**Affiliations:** 1 Division of Rheumatology, University of Southern California Keck School of Medicine, Los Angeles, USA

**Keywords:** access to health care, healthcare disparity, immune checkpoint inhibitors (icis), immune-related adverse event (irae), patient referral

## Abstract

Introduction: Immune checkpoint inhibitors (ICIs) have revolutionized cancer therapy; however, they have been associated with clinically significant immune-related adverse events (irAEs), including rheumatic irAEs. Rheumatic irAEs due to ICIs are not well characterized among ethnic minorities. Objective: Accordingly, we investigated rheumatic irAEs and referrals to rheumatologists at a public medical center (MC) serving a predominantly Hispanic population and a neighboring private MC serving a predominantly a population that identified as White, Black, Asian, or Indigenous population, with each MC being staffed by faculty from the same medical school. Methods: The medical records of patients 18+ years seen at either MC from May 2015 through May 2021 were screened for treatment with ICIs by ICD codes. We compared characteristics of ICI-treated patients who developed irAEs vs. those who did not develop irAEs. Overall, 28 (20.9%) and 74 (15.5%) patients developed documented irAEs at the public and private MCs, respectively. Of these, 21 patients (six at the public MC; 15 at the private MC) developed rheumatic irAEs. We compared referral rates to rheumatologists and management of rheumatic irAEs between these two groups. Results: Across both MCs, the most common rheumatic irAEs were arthritis (66.7%) and myositis (14.3%). Compared to private patients, public patients were more likely to be Hispanic (66.7% vs. 13.3%, p=0.012) and less likely to be referred to a rheumatologist (16.7% vs. 66.7%, p=0.036). There were no significant differences between institutions in cancer outcomes in patients with rheumatic iRAEs. Conclusion: The significantly lower rate of referrals to rheumatologists among patients with rheumatic irAEs at a public MC compared to patients at a neighboring private MC raises concern for healthcare disparities. Larger studies with racially diverse patients are necessary to help ensure equitable care for all patients.

## Introduction

The institution of immune checkpoint inhibitors (ICIs) has revolutionized cancer therapy [1]. However, ICI therapy is associated with clinically significant immune-related adverse events (irAEs), likely due to non-tumor-specific immunological activation [2]. irAEs may affect multiple organ systems, with the median time to onset of irAEs estimated at around 38 weeks, with onset varying from one to 127 weeks [3]. Rheumatic irAEs, which represent a broad spectrum of clinical syndromes including arthritis, myositis, vasculitis, scleroderma, sicca syndrome, and others, are not uncommon and have attracted considerable attention in recent literature [1]. The prevalence of rheumatic irAEs has been reported as 2.8% in one large study [4] and at 5% to 10% in other studies [5], although the true prevalence may be greater due to underreporting of cases.

Understanding ICI-related rheumatic irAEs has become increasingly important to oncologists, as they are the ones who identify the ICI-related toxicities, and to rheumatologists, who are in the best position to manage and treat the rheumatic irAEs. Timely treatment of rheumatic irAEs is essential not only to prevent long-term complications such as erosive joint damage but also to maintain functional capacity and quality of life [6]. This is particularly important as rheumatic irAEs may persist for even years after cessation of ICI therapy [7]. To date, the majority of patients included in studies of rheumatic irAEs are White, Black, Asian, or Indigenous populations [2] and are, therefore, not representative of all patient populations, especially those in urban settings.

Moreover, the frequency, severity, and treatment of rheumatic irAEs are not well characterized in racial and ethnic minorities. This is not surprising, as several studies have demonstrated the underrepresentation of ethnic minorities in rheumatology research. A systematic review by Strait et al. [8] of rheumatoid arthritis patients enrolled in 126 randomized clinical trials (RCTs) between 2008 and 2018 found that enrollment of minority ethnic groups was significantly lower than their representation within the US Census population (16% vs. 40%; p < 0.001). Such underrepresentation was also seen in clinical trials for patients with systemic lupus erythematosus (SLE), with a review by Falasinnu et al. [9] finding that Black patients only represent 14% of RCT enrollees despite encompassing 43% of SLE cases. These disparities may be linked to several factors, including patients’ access to clinical trials, cultural factors, and whether the institutions at which patients receive care are public vs. private. 

Given the frequent differences in race/ethnicity between “public” patients and “private” patients, coupled with any implicit biases that may exist among treating physicians, we hypothesized that healthcare disparities between “public” and “private” patients may impact rheumatic irAE management and referral rates to rheumatologists. Accordingly, we investigated the nature of rheumatic irAEs among patients treated with ICIs and subsequent referrals to rheumatologists at two medical centers (MCs) (Los Angeles General Medical Center (LAG), a safety-net public hospital, and Keck Medical Center (KMC), a private hospital) located in the same geographic region of Los Angeles, CA, each staffed by faculty from the same medical school.

This article was previously presented as a meeting abstract at the American College of Rheumatology Annual Scientific Sessions (Convergence) on November 13, 2023. 

## Materials and methods

The electronic medical records (EMRs) of patients 18+ years of age seen at LAG and KMC from May 2015 through May 2021 were screened for a diagnosis of malignancy and treatment with ICIs (nivolumab, pembrolizumab, atezolizumab, ipilimumab, cemiplimab, avelumab, durvalumab, or combination therapy) by International Classification of Diseases, 9th Revision (ICD-9) codes [10] (May 2015-September 2015) and ICD-10 codes [11] (October 2015-May 2021). The timeframe from 2015 to 2021 was chosen to reflect the time period during which ICIs were broadly adopted in clinical practice and allowed us to capture a sufficient number of patients who were treated with different ICIs. Retrospective review of medical records was approved by the University of Southern California Institutional Review Board (approval number: HS-20-00831). Data, including demographics (age, gender, ethnicity), malignancy type, and comorbidities, were collected. Patients with a history of pre-existing rheumatologic disease were excluded. To minimize misclassification bias, each patient's chart was reviewed by an investigator with a background in rheumatology (rheumatology fellow and/or attending physician) to verify the administration of ICI, patient diagnosis of cancer, and the presence of ICI-related rheumatic irAE. Charts were vetted for documented rheumatic irAEs, including arthritis, sicca syndrome, myositis, SLE-like features, and others, by the caring physicians (oncologists, primary care physicians, and/or consulting rheumatologists).

The diagnosis of rheumatic irAE was determined by review of clinical, laboratory, and imaging findings, with fulfillment of American College of Rheumatology (ACR) [12] and/or European Alliance of Associations for Rheumatology (EULAR) criteria [13] where applicable. We assessed whether patients received steroid monotherapy, non-steroidal anti-inflammatory drugs (NSAIDs), steroids plus NSAIDs, disease-modifying antirheumatic drugs (DMARDs), opioids, or no treatment for their rheumatic irAEs. In addition, we evaluated whether ICI therapy was continued or stopped due to the presence of the rheumatic iRAEs. For those patients who received treatment for rheumatic iRAEs, we evaluated whether or not there was complete or partial resolution of the rheumatic iRAE following treatment. We compared demographics, referral rates to a rheumatologist, and management of rheumatic irAEs among patients with rheumatic irAEs at each institution. Chi-squared testing and T-testing were used for analyses, with P < 0.05 considered statistically significant.

## Results

We identified 611 individual patients (134 at LAG and 477 at KMC) with a malignancy who were treated with ICIs. Of these, 28 (20.9%) LAG patients and 74 (15.5%) KMC patients developed irAEs as documented by the treating oncologists and/or the consulting rheumatologists. No significant differences in demographics and comorbidities were detected between the irAE and no-irAE groups at either institution (Table 1).

**Table 1:** Characteristics Among Patients at LAG and KMC with irAEs vs. No irAEs LAG: Los Angeles General; KMC: Keck Medical Center; irAE: immune-related adverse event; ICI: immune-checkpoint inhibitor Pulmonary: small cell lung cancer, pulmonary carcinoid tumors; *Other: unknown primary (5), adrenal (1), anal (5), basal cell carcinoma (2), cardiac angiosarcoma (1), cervical (11), cholangiocarcinoma (1), chordoma (1), central nervous system (CNS) lymphoma (1), colon (8), cutaneous squamous cell carcinoma (SCC) (1), cystic carcinoma maxillary sinus (1), desmoplastic round small cell tumor (1), duodenal (1), esophageal (5), follicular lymphoma (1), Hodgkin lymphoma (6), neuroendocrine (4), perivascular epithelioid cell (1), maxillary sinus SCC (1), Merkel cell (1), mesothelioma (4), thyroid (3), mucoepidermoid (1), nasopharyngeal (4), nerve sheath tumor (1), osteosarcoma (1), Pancoast tumor (1), pancreatic (1), penile (1), rectal (3), cutaneous SCC (3), sarcoma (7), fallopian tube (1), vulvar (2), testicular (1), thyroid (1), thymic (2), prostate (4), multiple cancers (48)

Characteristic	LAG irAEs (n=28) n(%)	LAG No irAEs (n=106) n (%)	KMC irAEs (n=74) n(%)	KMC No irAEs (n=403) n(%)
					Age at Cancer Diagnosis (years ± SD)	56.4 ± 11.5	57.1 ± 11.8	60.7 ± 13.9	62.2 ± 13.1
					Age at Initiation of ICI Treatment (years ± SD)	58.9 ± 10.6	59.4 ± 11.4	64.0 ± 13.4	65.5 ± 12.5
Sex				
Male	13 (46.4)	60 (56.6)	38 (51.4)	248 (61.5)
Female	15 (53.6)	46 (43.4)	36 (48.6)	155 (38.5)
Ethnicity				
Hispanic	16 (57.1)	69 (65.1)	12 (16.2)	88 (21.8)
White, Black, Asian, or Indigenous	12 (42.9)	37 (35.9)	62 (83.8)	311 (77.2)
Unknown	0 (0)	0 (0)	0 (0)	4 (1.0)
Type of Cancer				
Melanoma	5 (17.9)	13 (12.3)	12 (16.2)	35 (8.7)
Non-small Cell Lung Cancer	7 (25.0)	27 (25.5)	6 (8.1)	44 (10.9)
Renal	4 (14.3)	14 (13.2)	15 (20.3)	74 (18.4)
Urothelial	0 (0)	7 (6.6)	2 (2.7)	32 (7.9)
Pulmonary*	0 (0)	7 (6.6)	4 (5.4)	40 (9.9)
Breast	0 (0)	1 (0.9)	1 (1.4)	11 (2.7)
Oral Squamous Cell	0 (0)	3 (2.8)	1 (1.4)	13 (3.2)
Ovarian	0 (0)	0 (0)	4 (5.4)	7 (1.7)
Endometrial	2 (7.1)	5 (4.7)	4 (5.4)	13 (3.2)
Hepatocellular	1 (3.6)	4 (3.8)	2 (2.7)	24 (6.0)
Gastric	1 (3.6)	2 (1.9)	1 (1.4)	14 (3.5)
Other**	8 (28.6)	23 (21.7)	22 (29.7)	96 (23.8)
Comorbidities				
Hypertension	11 (39.3)	45 (42.5)	37 (50.0)	202 (50.1)
Obesity	6 (21.6)	15 (14.2)	25 (33.8)	96 (23.8)
Hyperlipidemia	10 (35.7)	27 (25.5)	12 (16.2)	93 (23.1)
Asthma	1 (3.6)	0 (0)	3 (4.1)	10 (2.5)
End-Stage Renal Disease	0 (0)	2 (1.9)	0 (0)	13 (3.2)
Chronic Obstructive Pulmonary Disease	1 (3.6)	3 (2.8)	1 (1.4)	25 (6.2)
Congestive Heart Failure	2 (7.1)	7 (6.6)	2 (2.7)	11 (2.7)
Diabetes	7 (25.0)	32 (30.2)	12 (16.2)	91 (22.6)
Chronic Kidney Disease	8 (28.6)	11 (10.4)	7 (9.5)	42 (10.4)

Of the 102 patients with documented irAEs, 21 (six LAG + 15 KMC), representing 3.4% of the ICI-treated patients in our combined cohort, developed rheumatic irAEs. Across both MCs, the most common rheumatic irAEs were arthritis (66.7%), followed by myositis (14.3%). Other identified rheumatic irAEs included sicca syndrome (4.8%), Achilles tendonitis (4.8%), De Quervain’s tenosynovitis (4.8%), and SLE-like features (4.8%) (Table 2). 

**Table 2:** Demographics Among Patients with Rheumatic iRAEs LAG: Los Angeles General; KMC: Keck Medical Center; irAE: immune-related adverse event; ICI: immune-checkpoint inhibitor; SLE: systemic lupus erythematosus

Characteristic	Patients with Rheumatic irAEs at LAG (n=6) n(%)	Patients with Rheumatic irAEs at KMC (n=15) n(%)	P-value
				Age at Cancer Diagnosis (Years ± SD)	54.7 ± 9.3	58.4 ± 14.0	0.557
Age at Initiation of ICI Treatment (Years ± SD)	56.8 ± 8.2	59.9 ± 13.4	0.606
Sex			0.577
Male	2 (33.3)	7 (46.7)	
Female	4 (66.7)	8 (53.3)	
Ethnicity			0.012
Hispanic	4 (66.7)	2 (13.3)	
White, Black, Asian, or Indigenous	2 (33.3)	13 (86.7)	
Type of rheumatic iRAE			0.288
Arthritis	3 (50.0)	11 (73.2)	
Sicca Syndrome	0 (0)	1 (6.7)	
Myositis	2 (33.3)	1 (6.7)	
Achilles Tendonitis	0 (0)	1 (6.7)	
De Quervain’s Tenosynovitis	0 (0)	1 (6.7)	
SLE-like Features	1 (16.7)	0 (0)	

**Figure 1:**
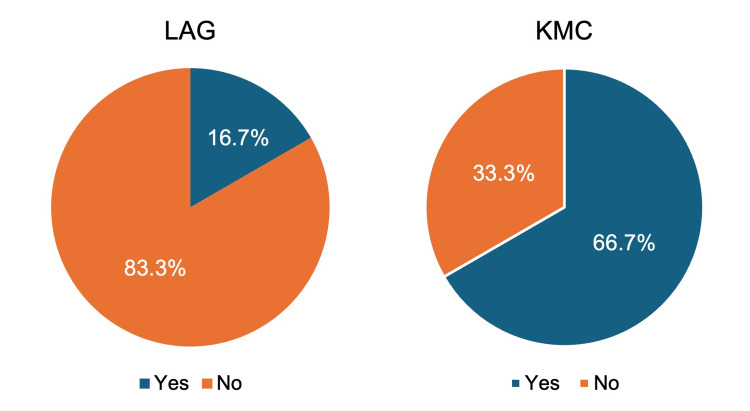
Percentage of Patients Referred to Rheumatology LAG: Los Angeles General; KMC: Keck Medical Center

Compared to patients at KMC, patients at LAG with rheumatic irAEs were more likely to be Hispanic (66.7% vs. 13.3%, p=0.012) (Table 2) and less likely to be referred to a rheumatologist (16.7% vs. 66.7%, p=0.036) (Figure 1).

**Figure 2:**
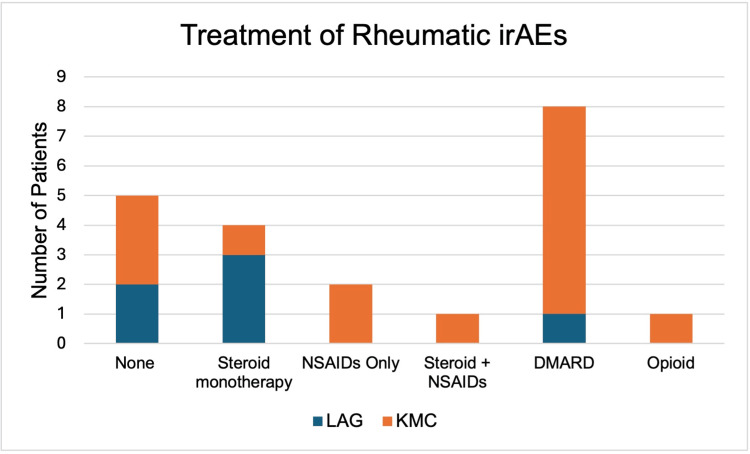
Treatment of Rheumatic irAEs irAEs: immune-related adverse events; NSAIDs: non-steroidal anti-inflammatory drugs; DMARD: disease-modifying antirheumatic drug; LAG: Los Angeles General; LAG: Los Angeles General; KMC: Keck Medical Center

The majority of patients at LAG and KMC (66.7% and 66.7%) continued ICI treatment despite the development of rheumatic irAEs. In addition, most patients at LAG and KMC (66.7% and 86.7%) had partial or complete resolution of rheumatic irAEs following treatment. There were no significant differences in the treatment of rheumatic irAEs among patients at LAG and KMC (Figure 2). 

In addition, there were no significant differences in rheumatic irAEs outcomes or cancer outcomes, including complete or partial response, stable disease, progression, or death, between patients with rheumatic irAEs at the individual institutions (Table 3). 

**Table 3:** Management and Outcomes of Rheumatic iRAEs LAG: Los Angeles General; KMC: Keck Medical Center; irAE: immune-related adverse event; ICI: immune-checkpoint inhibitor; NSAIDs: non-steroidal anti-inflammatory drugs; DMARD: disease-modifying antirheumatic drug

Characteristic	Patients with Rheumatic irAEs at LAG (n=6) n(%)	Patients with Rheumatic irAEs at KMC (n=15) n(%)	P-value
				Referred to a Rheumatologist	1 (16.7)	10 (66.7)	0.036
Treatment of irAE			0.245
None	2 (33.3)	3 (20.0)	
Steroid Monotherapy	3 (50.0)	1 (6.7)	
NSAIDs Only	0 (0)	2 (13.3)	
Steroid + NSAIDs	0 (0)	1 (6.7)	
DMARD	1 (16.7)	7 (46.6)	
Opioid	0 (0)	1 (6.7)	
ICI Treatment			1
ICI Stopped	2 (33.3)	5 (33.3)	
ICI Continued	4 (66.7)	10 (66.7)	
Cancer Outcome			0.612
Complete Response	1 (16.7)	1 (6.7)	
Partial Response	1 (16.7)	1 (6.7)	
Stable	2 (33.3)	4 (26.6)	
Progression	2 (33.3)	6 (40.0)	
Death	0 (0)	3 (20.0)	
Rheumatic irAE Outcome			0.292
Complete or Partial Resolution After Treatment	4 (66.7)	13 (86.7)	

## Discussion

This study took advantage of the unique position of our Division of Rheumatology as the sole providers of all rheumatology services at both a private MC (KMC) and a public MC (LAG) in the same geographic region. In our study, the prevalence of rheumatic irAEs of 3.4% among patients treated with ICIs is consistent with reported prevalence in the literature [14]. In our study, arthritis represented the great majority (66.7%) of rheumatic irAEs, similar to the findings of others [15]. The rheumatic irAEs were usually mild, and most of the affected patients (66.7% across both institutions) were continued on ICI therapy. Strikingly, our study identified a significant difference in the rate of referrals to rheumatologists at LAG compared to KMC (16.7% vs. 66.7%, p=0.036). The discrepancy in referral rates among physicians who refer patients to rheumatology is a disconcerting reflection of healthcare disparities that may exist between the two institutions, despite being staffed by the same faculty from the same medical school. A greater percentage of the studied LAG patients were of Hispanic ethnicity than the studied KMC patients, and, from anecdotal experience, were also more likely to be Spanish-speaking only. Across the entire Los Angeles County Health Services system, which includes LAG, over 1.2 million Spanish-preferred patient visits occur yearly, which accounts for about 50% of total visits. Moreover, approximately 93% of non-English-speaking patients at LAG prefer Spanish [16]. Therefore, we suspect that the decreased referral rate of LAG patients to rheumatologists is likely multifactorial and could include language barriers, access to care, and implicit biases among faculty members. A review by Cappelli et al. [17] describes the challenges that arise in referrals to rheumatologists for suspected rheumatic irAEs, including the unfamiliarity of non-rheumatology providers with the musculoskeletal examination to detect subtle abnormalities. This may be more significant in older patients with cancer who may have comorbidities such as osteoarthritis that confound the clinical picture. While these challenges may be true, they do not explain the disparity between referral rates at our public vs. private institutions, pointing to implicit bias possibly playing a role in differential referral rates among different patient populations. Given the emergence of rheumatic irAEs and the importance of establishing early care with rheumatologists to facilitate optimal management, the EULAR has published principles to guide the diagnosis and management of rheumatic irAEs [18]. Currently, referral algorithms are based on the severity of irAEs based on the Common Terminology Criteria for Adverse Events (CTCAE). The first international guidelines recommended referral to a rheumatologist in the case of severe symptoms not responding to glucocorticoids (grade 3) [18]. However, the CTCAE grading scale is less commonly used by rheumatologists than by oncologists, does not always accurately reflect the severity of rheumatologic disease, and may be variable due to the subjective nature of the grading scale by treating physicians. Therefore, EULAR has recommended prompt referral to rheumatologists prior to initiation of glucocorticoids [18]. In addition, the American Society of Clinical Oncology (ASCO), in collaboration with specialists including rheumatologists, has published guidelines on the management of organ-specific irAEs, including rheumatic irAEs [6]. The guidelines address management of rheumatic irAEs, including inflammatory arthritis, polymyalgia-like syndrome, and myositis. Regarding inflammatory arthritis, ASCO recommends early referral to a rheumatologist if there is joint swelling (synovitis) or if symptoms persist for more than four weeks. Management strategies include treatment with NSAIDs for mild inflammatory arthritis, with consideration of prednisone if no improvement, and intra-articular steroid injections for large joints, if affected. While corticosteroids may be used, the guidelines caution against prolonged use and recommend steroid-sparing agents, including DMARDs, in cases refractory to initial therapy. Overall, the guidelines stress the importance of prompt referral to rheumatologists in order to expedite diagnosis and initiate targeted therapy [6]. Despite published guidelines, available data regarding referral rates to rheumatologists suggest that referrals are not common and may be associated with a delay in diagnosis of rheumatic irAEs. A retrospective study by Liew et al. [19] found that only four of 12 patients (33.3%) experiencing rheumatic irAEs were evaluated by rheumatologists. The authors hypothesized that in clinical practice, many rheumatic irAEs may be mild in nature and, therefore, not referred to rheumatologists for evaluation and management. In contrast, a study by Lidar et al. [15] that evaluated patients treated with ICIs at a large tertiary cancer center found that 12 of 14 patients (85.7%) with rheumatic irAEs were referred to rheumatologists for further evaluation. Of note, the majority of rheumatic irAEs (92.9%) in this study were graded as either moderate or severe using the CTCAE grading scale, a possible explanation for the higher rate of referrals compared to other studies [15]. The authors of this study proposed a series of standardized screening questions for oncologists when assessing possible rheumatic irAEs to help facilitate appropriate referrals to rheumatologists [15]. A retrospective review by Pacholczak-Madej et al. [20] recommended referral to rheumatologists if no improvement in symptoms after initial treatment with NSAIDs, even in the case of mild symptoms, including CTCAE grade 1 rheumatic irAEs. In addition, Melissaropoulos et al. [21] suggest a multidisciplinary approach in which oncologists periodically screen patients receiving ICIs for musculoskeletal symptoms and refer them to rheumatologists if patients experience persistent symptoms. Identifying risk factors for the development of rheumatic irAEs may prove to be helpful in detecting symptoms early. A retrospective study by Cunningham‐Bussel et al. [4] of 226 patients with rheumatic irAEs who were treated with ICIs found that baseline predictive factors for the development of rheumatic irAEs include melanoma and genitourinary cancer, combination ICI treatment, and preexisting autoimmune disease. Another study by Pundole et al. [22] found that rates of rheumatic irAEs were higher in men and in patients older than 65 years. These studies suggest that recognizing patients who are at higher risk may facilitate earlier diagnosis and more prompt referral to rheumatologists [4]. Limitations of our study include the small sample size at each institution and the retrospective nature of our study, which permitted detection of irAEs only through chart review. As a result of the retrospective design, underreporting of irAEs may have occurred. Prospective studies to confirm our findings are urgently needed. Clinical interpretation of results was also limited as we did not specifically examine time to referral to rheumatology, symptom severity at presentation, or functional impact of rheumatic irAEs. In addition, we did not adjust for therapeutic advances (i.e., evolving ICI regimens) during our six-year study timeframe that may have impacted treatment strategies for rheumatic irAEs. Future studies that evaluate rheumatic irAEs by different treatment eras may be helpful to evaluate for differences in therapy over time. Addressing this issue will require future studies that include multiple centers with racially, ethnically, and socioeconomically diverse patients. In addition, analyses were limited to available data from chart review, as our statistical analysis did not account for missing data. However, missing data were minimal for most patients, so the impact on our results was also likely minimal. Finally, we did not perform multivariate regression models to adjust for confounding factors (i.e., age, cancer type, comorbidities) due to our small sample size. Future studies with larger cohorts of patients are needed to adjust for potential confounding factors. 

## Conclusions

In conclusion, our study points to a disconcerting disparity in referrals to rheumatologists for rheumatic irAEs between ICI-treated patients at a public institution and ICI-treated patients at a private institution. Efforts are needed to reduce such discrepancies, which could include not only improved collaboration and communication between oncologists and rheumatologists but also greater awareness of latent healthcare disparities. In addition, institutional strategies to mitigate referral disparities include referral algorithms for patients on ICIs who develop rheumatologic symptoms, automatic rheumatology consult triggers in the EMR system based on patient diagnoses (i.e., new arthritis while on ICI), and the development of standardized screening questions for oncologists when assessing possible rheumatic irAEs. Additional prospective, multi-center studies, especially among racial and ethnic minorities, are needed to better our understanding of how to dismantle barriers to care for our underserved populations.
